
JOURNAL INFORMATION
==============================
NLM Title Abbreviation: J Child Orthop
Journal ID: J Child Orthop
NLM Journal ID: 101313582

Journal of Children's Orthopaedics

ISSN: 1863-2521
EISSN: 1863-2548
Publisher: SAGE Publications

ARTICLE INFORMATION
==============================
PMCID: PMC4794450
DOI: 10.1007/s11832-016-0720-1
Article ID: 720
Article version: 1
Subjects: Author Index

Author Index EPOS, 2016, Rome, Italy

Electronic publication date: 2016 Mar 16
Print publication date: 2016 Apr
Volume: 10
Issue: Suppl 1
First page: 1
Last page: 7
Copyright: © The Author(s) 2016
License: This article is distributed under the terms of the Creative Commons Attribution 4.0 International License (http://creativecommons.org/licenses/by/4.0/), which permits unrestricted use, distribution, and reproduction in any medium, provided you give appropriate credit to the original author(s) and the source, provide a link to the Creative Commons license, and indicate if changes were made.
License URL: https://creativecommons.org/licenses/by/4.0/

issue-copyright-statement: © The Author(s) 2016

==============================

Abbas Naqvi, Gohar OP78
Abdelaal, Ahmed OP97
Abid, Aziz OP137
Abitan, Alexandre OP57
Abouel-Enin, S OP51
Abousamra, Oussama OP101 OP82 OP113 EP43 OP78 OP109 EP58
Abril, Juan Carlos EP66
Accadbled, Franck OP38 EP83
Adams, Mark PP4 OP72
Agneray, Hélène OP134
Aguirre Canyadell, Màrius OP87
Ahn, Tae Young OP86
Ahn, Hyeon Wook EP26
Akceylan, Anil EP24
Akpinar, Erhan OP64
Aksahin, Ertugrul EP3
Al Khouri Salem, Hassan EP90
Al Khudairy, Ammar EP18
Al-Naser, Saeed OP140
Alameldin, Mohamed OP97
Alexiev, Venelin PP7
Alriksson Schmidt, Ann EP23
Alves, Alexandra OP70
Alves, Cristina OP95 OP40 EP96 EP94 EP102
Alzahrani, Mohammad EP36
Amares, Chavarin EP13
Amelia, MD, Marielle OP84
Ammann, Elias OP114
Andras, Lindsay OP71 OP62 OP28 OP63
Andras, MD, Lindsay OP25
Andreacchio, Antonio EP20 OP24
Anne-Laure, SIMON EP82
Anne-Laure, Simon OP81
Anoua, Nikolaos PP15
Antoniadou, Thekla OP76
Aquilani, Alex OP117
Arbucci, John OP139
Arkader, Alexandre OP34 OP120 OP28
Armenter Duran, Júlia OP32
As-Sultany, Mohamed EP105
Asghar, Jahangir OP59
Assaghir, Yasser OP97
Assi, Ayman EP56 OP66 OP129 EP54
Åström, Eva OP83
Attwenger, Bernhard OP3
Aulisa, Angelo Gabriel OP68
Aulisa, Angelo EP76
Aydemir, Ahmet Nadir OP37 OP35

Backteman, Torsten OP45
Baindurashvili, Alexey OP20 OP123
Bakouny, Ziad EP54 EP56 OP66
Balacó, Inês EP96 EP94 OP40 OP95 EP102
Balasubramanian, Balakumar OP51 EP74
Barbaro, Daniele PP9 PP10
Barbary, Stephane OP14
Barbier, Dominique OP39 EP83
Barla, Manuela OP14
Bartonek, Åsa EP52
Bascom, Nathan EP37
Bat'kin, Sergey PP11
Bauer, Jennifer EP29
Baumgart, Rainer OP15 OP89 EP89
Baunin, Christiane OP38
Beaty, MD, James EP98
Beek, Erik OP43
Bellini, Diego EP39
Benesch, Thomas EP77
Bennis, Mehdi EP83
Berger, Leopold EP55
Bergerault, Francois OP91
Bernard, Louis OP41
Bernardi, GABRIELE EP2
Bernius, Peter OP100
Bertana, Sandra OP130
Besselaar, Arnold OP43
Bessems, Gert EP91
Beunder, Tom EP91
Bicimoglu, Ali EP3
Bilili, Eva OP80
Bilnicki, Tadeusz EP57
Birke, Oliver EP68 OP44
Bizdikian, Aren Joe OP66
Björk, Lars EP53
Black, Sheena OP11
Blackey, Caroline M OP51
Blanco, John OP79 OP50 OP126
Bland, Daniel OP11
Blumetti, Francesco OP106 OP103 EP5
Böhm, Harald OP128
Bollini, Gérard OP60
Bollini, Gerard OP134
Bonetti, Ingrid PP16
Bonnard, Christian OP91 OP41

Bor, Noam OP55
Borgo, Andrea OP130
Bork, Daniel EP77
Botnari, Alexei OP69
Brandolini, Anna PP9 PP10
Breyer, Sandra R. OP141
Brice, Ilharreborde OP81 EP82
Brites Moita, Miguel OP40
Broom, Alexander OP28 OP34
Broom, BA, Alexander OP25
Brouchet, Anne OP38
Brown, Elliott EP4
Bruce, Colin EP86
Brunner, Reinald OP114
Buckingham, Rachel EP48
Buklaev, Dmitry PP11
Burger, Lisebette OP133
Burger, Bart OP133
Butterfiled, Matthew EP87
Buxbom, Peter EP72
Buyukdogan, Kadir OP64
Bytyqi, Cen EP104

Cabello, Juan OP22
Cabello Blanco, Juan EP33
Caird, Michelle OP119
Caliskan, Emrah OP37 EP10
Camathias, Carlo OP115 OP114
Canavese, Federico OP24 EP20 OP69
Carbone, Marco OP125
Carbonell, Rafael EP32
Carender, Christopher N. OP58
Carrothers, Andrew OP77
Carter, Cordelia EP4
Carvalho, Alice OP40 OP95
Castelein, René OP43
Catterall, Anthony PP8
Cédric, Baumann OP1
Ceroni, Dimitri OP42 EP12 OP32 EP36
Cersosimo, Antonella OP130
Chabrand, Patrick EP11
Chan, Priscella EP51 OP63
Chan, Cheri Su Hui OP73
Chen, Cynthia OP50
Chen, Andrew EP27
Chen, Brian Po-Jung OP109
Chen, Cynthia OP126
Cheon, Jung-Eun OP94
Cherkashin, Alexander OP5
Chiang, Ming-Hung OP124
Chiari, Catharina EP77
Cho, Tae-Joon OP94
Choi, In Ho OP94
Choi, Paul EP51 OP34 OP28 OP63 OP119 EP97
Chomiak, Jirí OP19
Choufani, Elie OP60
Christodoulou, Michael OP76 OP46
Christov, Boyan PP7
Chu, Alice EP60 EP59
Chua, Chong Bing OP73
Chung, Myung Ki PP2
Chung, Chin Youb PP2
Church, Chris OP82 OP101
Claessen, Femke OP118
Clark, Zach EP21
Clarke, Nicholas OP47
Cobanoglu, Mutlu OP2
Cometto, Matteo OP111
Connor, Justin EP43 OP101 OP82
Conry, Keegan EP27 OP7
Cooke, Stephen OP116 OP117
Cooperman, Daniel EP8 OP138
Corbu, Andrei EP81
Cornwall, MD, Roger EP38
Cosgrove, Aidan EP70
Cosma, Dan EP81
Costici, Pier Francesco EP15
Crawford, Alvin H OP56
Crawford, Lindsay EP88
Crawford, MD, FACS, Alvin H. EP44
Cucchiaro, Giovanni OP71
Cullu, Emre OP2
Cyr, BA, Micaela OP25
Czuba, Tomasz OP102

d’ Heurle, Albert OP122
Dabney, Kirk EP43
Daher, Michel EP31
Danielewicz-Bromberek, Anna OP8
Danielsson, Aina OP45
Danino, Barry EP46
Dautel, Gilles OP39 OP14
Dayer, Romain EP36
de Céano-Vivas la Calle, María EP33
De Courtivron, Benoit OP91
de Cristo, Claudia EP45
De las Heras Sotos, Jorge EP47
De Luna, VINCENZO EP2
de Morais Filho, Mauro C OP106 OP103 EP5
De Pellegrin, Maurizio EP16 EP28 PP17 OP93
de Poorter, Jolanda EP91
de Vos-Jakobs, Suzanne OP118
Dede, Ozgur OP64
Demirkiran, Gokhan OP64
Denning, Jamie R EP40 EP37
Derienne, Jospeh EP31
Destombes, Louise OP38
Di Carlo, Valentina OP125
Dibello, Daniela OP125
Diene, Gwanaelle OP137
Dierauer, Stefan EP67
Dimeglio, Alain OP69
Dimitriadis, Dimitris OP112
Dixon, Philippe OP4
Dodwell, Emily OP126 EP62 OP79 OP23 OP50
Doederlein, Leonhard OP128
Dohin, Bruno EP90
Donnelly, Kevin EP70
Dormans, John OP59
Dowling, Jamie OP53
Doyle, Shevaun OP50 OP79 OP126
Dragoni, Massimiliano EP39
Duart Clemente, Julio EP66
Dubois Ferriere, Victor OP32
Dubousset, Jean OP69
Dungl, Pavel OP19
Duque Orozco, Maria Pilar OP101 OP82 EP43 OP113 OP78 OP109 EP58
Dussa, Chakravarthy U OP128
Dzik, Marek EP57 EP61

Eamsobhana, Perajit EP79
Eastwood, Deborah OP135
Eberhardt, Oliver EP73 OP18 OP33 OP54 EP103
Edmonds, MD, Eric OP84
Eichler, Johannes EP55
Eidelman, Mark OP30 OP55
Eisman, Emily A OP56
Ekholm, Catarina OP104
El-Hawary, Ron OP61 EP18 EP63
Eliasson, Ann-Christin OP104
Elliott, Marilyn OP31
Emans, John OP62
Encisa, Clarissa EP78
Engesaeter, Ingvild O OP52
Engesaeter, Lars B OP52
Eriksson, Marie EP52
Ernat, Justin OP26 OP27
Erol, Bulent OP37 OP35 EP10
Ersoz, Murat EP3
Eygendaal, Denise OP118

Falciglia, Francesco OP68 EP76
Farr, Sebastian PP1 OP3
Farrington, David OP75
Farsetti, PASQUALE EP2
Farsetti, Pasquale EP39
Fatyga, Marek OP8
Faulks, Shawne EP64 OP132
Fawi, Hassan EP105
Feldman, David EP97
Feldman, Oren OP30
Feldman, David EP59 EP60
Felton, Kevin OP92 EP64
Felus, Jaroslaw OP96
Fenoglio, Amy EP4
Fenstad, Anne Marie OP52
Fernandes, James A OP48
Fernandes, James OP140
Fernandez, Francisco OP54 OP33
Fernandez, Francisco F EP103
Fernandez Ansorena, Ana EP66
Fernandez Fernandez, Francisco OP18 EP73
Fernández Polo, Aurora OP9
Ferrer Santacreu, Eugenio EP47
Fischerauer, Stefan EP55
Fletcher, Nicholas EP18
Florence, Müller OP1
Flournory, BA, Jolecia OP127
Flynn, John OP28
Flynn, Tara OP61
Flynn, John OP34
Flynn, John (Jack) EP63
Flynn, MD, John OP25
Fontecha, César G OP87
Fontecha, Cesar G OP9
Forni, Fabio OP130
Forriol Campos, Francisco OP12
Fracassetti, Dario PP17 OP93 EP28
Frost, Charlotte OP108
Frydrychová, Monika OP19
Fujino, Marcelo OP106 OP103 EP5
Fullarton, Mairi EP48
Fung, Winnie Kar Yee OP73

Galban, Miguel OP142
Galla, Ambra PP10 PP9
Ganger, Rudolf OP3
Gannon III, BS, Edwin EP98
Gantelius, Stefan EP53
Garcia-Mata, Serafin EP66
Gareb, Barzi EP91
Garg, Neeraj EP86
Garg, MD, Sumeet OP25
Gargan, Martin OP49
Garin, Christophe OP134
Garriguez Pérez, Daniel EP33
Gaulthier, Luke EP18
Gavaret, Martine OP60
Gebhart, Jeremy EP8 OP7
Gelman, Yechiel EP75
Genovesio, Cécile EP11
Gera, Sumanth EP30
Ghanem, Ismat EP31 OP66 EP56 OP129 EP54
Gibbons, Paul OP44
Gibson, Peter OP72 PP4
Gilabert Carbajo, Carla OP9
Giles, Stephen OP140
Gilmore, Allison EP65 EP19 EP27 OP138
Giordano, Marco EP76 OP68
Girsch, Werner PP1
Glorion, Christophe OP70
Godfrey, Jenna OP119 EP51
Godfried, David EP97
Goldman, Vladimir EP75
Goldstein, Rachel EP51 EP49 OP107 EP50 EP42
Goldstein, Rachel Y OP62 OP71 OP120
Goldstein, Rachel EP41
Goliana, Sergei OP20
Gonzalez, Amanda EP36
González López, Juan José OP9
Gonzalez-Moran, Gaspar OP22 EP32
Gornitzky, Alex OP34
Gosens, Taco OP118
Gosheger, Georg OP17
Gould, Heath EP101
Graham, Kerr OP108
Grammatico-Guillon, Leslie OP41
Grazioli, Aron OP32
Green, Daniel W OP139
Green, Daniel OP79 OP23 OP50
Gremark, Ola EP53
Grill, Franz EP46
Grimberg, Dominic EP19
Guindani, Nicola EP103
Guzzanti, Vincenzo OP68 EP76

Haddad, Elie EP90
Hagglund, Gunnar EP71
Hägglund, Gunnar EP23 OP102
Hahne, Julia OP3
Halvorsen, Vera OP52
Han, Joon Woo OP105
Hanan, Liran EP1
Hardesty, Christina EP65
Harris, Liam OP28 OP62
Hayashi, Katsuhiro OP97
Hedequist, Daniel OP59
Heflin, John OP61
Heinsch, MD, David OP127
Helenius, Ilkka OP65 OP59
Henley, John OP101 OP82
Herngren, Bengt EP71
Herrera Soto, Jose EP87 EP88
Herrmann, Jochen OP141
Herzenberg, John E. EP46
Hines, Adam OP31
Ho, Michelle EP97
Ho, Christine OP26 OP27 OP121
Hollern, MD, Douglas A. EP44
Holmdahl, Per OP45
Honsawek, Sittisuk EP13
Hopkins, MD, Christopher EP98 EP99
Horn, Bernard David EP97
Horn, Joachim OP90
Horn, Anria OP135
Horn, Joachim EP14
Hosny, Gamal OP13
Houdek, Matthew OP110
Hovius, Steven OP133
Hsu, Angela EP51
Huber, Hanspeter EP67
Huhnstock, Stefan OP90 PP8
Hwang, Jin Ho OP105
Hwang, Il Yeong PP14

Ibnoulkhatib, Aissa OP137
Iershov, Dmytro PP19 PP18
Igoumenou, Vasileios OP76 OP46
Ilharreborde, Brice OP67 OP57
Inaba, Yutaka PP3 PP5
Iosifidis, Michael EP92
Ippolito, Ernesto EP39
Ippolito, ERNESTO EP2
Isabelle, Clerc-Urmès OP1
Issa, Samir-Pierre OP67

Jain, MD, Viral V EP38
Jang, Woo Young OP94
Jenner, Edward OP117 OP116
Jo, ChanHee EP64
Jo, Byung Chae PP2
Jo, ChanHee EP21
Jo, Ahreum EP17

Jo, ChanHee OP132
Jochymek, Jirí EP95
Johansson, Gabriella OP77
Johnston, Charles OP92 OP62
Joiner, Elizabeth OP63
Jones, Alvin OP29
Jordan, Robert OP117 OP116
Josyula, Sowmya OP79
Joukhadar, Nadim OP61
Journeau, Pierre EP36 OP39 OP134 OP14
Jouve, Jean-Luc OP39 OP60
Juchler, Céline OP42
Judd, Julia PP6
Jung, Ki Jin PP2
Jung, Sung Taek Jung EP17
Jylli, Leena EP52

Kadhim, Muayad EP63
Kalakucki, Jaroslaw OP85
Kaminski, Adam EP57
Kang, Seungcheol PP14
Kapetanakis, Ioannis PP15
Kapetanovic, Edi EP4
Kärrholm, Johan OP45
Karska, Klaudia OP85
Karski, Jacek OP85
Kawamura, Cátia OP106 OP103 EP5
Kay, Robert EP50 OP107
Kehayov, Raytcho PP7
Kellenberger, Christian EP67
Kelly, MD, Derek EP98 OP84 EP99 OP127 EP22
Kenis, Vladimir OP123 OP131
Keshet, Doron OP55
Keskinen, Heli OP65
Kessler, Jeffrey EP41
Keyvan, Mazda EP82 OP81
Khalil, Nour OP66 EP54
Khmyzov, Sergiy PP18
Khmyzov, Sergey PP19
Khusainov, Nikita PP11
Khusainov, Ruslan OP123
Kilani, Merhez EP90
Kilimci, Figen Sevil OP2
Kim, Jung Ryul OP10
Kim, Hui Taek OP86
Kim, Jae Young PP14
Kim, Jung Ryul OP74
Kim, Hyun Woo OP105
Kim, Byung Jin EP17
Kim, Seung Myun EP17
Kim, Min Cheol EP17
Kiran, Manish EP86
Kiriakidis, Anastasios EP92
Kissinger, Cathy OP71
Kleine-Koenig, Marie-Theres OP17
Knapik, Derrick OP138
Knapp, Raymond EP87 EP88
Koh, Mark Jean Aan OP73
Kolban, Maciej EP61 EP57
Koltsov, Andrey EP84
Konstantinou, Apostolos PP15
Korolkov, Oleksandr PP12
Köse, Nusret EP24
Kothari, Alpesh OP4
Kotlarsky, Pavel OP30
Koury, Kenneth OP72 PP4
Kowalczyk, Bart OP96
Krajbich, Joseph Ivan OP99
Kranzl, Andreas OP3
Kraus, Tanja EP55
Krauspe, Rüdiger EP93
Kroksmark, Anna-Karin EP52
Kubat, Ozren EP18 OP61
Kuldeep, Stohr OP77
Kummer, Fred EP59
Kuo, Ken N. OP124
Kurland, Adam EP60 EP59
Kutsikovich, MD, Jeffrey EP98 EP99
Kwon, Soon-Sun PP2

Lafage, Virginie OP66 EP54
Lakhal, Walid OP91
Lakomy, Jens PP13
Lalos, Charalampos PP15
Lamdan, Ron EP75
Lamont, Lauren EP21
Larrosa Escartín, María Nieves OP9
Larson, Noelle OP36
Latalski, Michal OP8
Laurence, Mainard-Simard OP1
Laurent, Emeline OP41
Lawniczak, Dominik OP48
Lebel, Ehud EP1
Lee, Kyoung Min PP2
Lee, In Hyeok PP2
Lee, Dong Hoon OP105
Lee, Nae Ho OP74 OP10
Leite, Jaqueline OP106 OP103 EP5
Lelkes, Valdis OP72
Lennon, Nancy OP82 OP101
Leveille, Lise OP5
Li, Ryan EP101 EP7
Lightdale, Nina OP120
Lim, Kevin OP73
Limpaphayom, Noppachart EP13
Lindberg, Antoinette OP119
Ling, Tah Pu EP102 OP95 OP40 EP94 EP96
Lirola, Jose EP66
Little, David OP44 EP68
Liu, Raymond EP65 EP19 OP21 EP7 EP101 EP27 OP138 EP8
Liu, Raymond W. OP58
Liu, Raymond OP6 OP7
Löffler, Jörg EP55
Lopes, José Augusto OP106 OP103 EP5
Lourenço, Alexandre EP6
Lovejoy, Steven EP29
Löwing, Kristina OP83
Lucenti, Ludovico EP45
Luderowski, Eva OP139

Maakaroun-Vermesse, Zoha OP41
Maathuis, Patrick EP91
Machida, Jiro PP3 PP5
MacKenzie, William OP59
Madan, Sanjeev OP51
Madan, Sanjeev S OP48
Madan, Sanjeev EP74
Maglione, Pierluigi EP39
Magnan, Bruno PP16
Mahadev, Arjandas EP30
Mallet, Cindy OP57
Mansour, Toni EP31
Mansour, Elie EP56
Mansour, Mounira OP69
Manzano, Sergio OP42
Marenco, Stefano EP20
Marengo, Lorenza OP24 EP20
Marich, Elena PP9 PP10
Markeas, Nikolaos OP46
Martí Seves, Ramon OP9
Marti-Ciruelos, Rafael EP66
Martin, Benjamin EP97
Martinelli, Elisabeth EP55
Martinez-Álvarez, Sergio EP36
Martins, Pedro Farinha EP96 OP95
Martus, Jeffrey EP29
Massaad, Abir OP66 EP54
Massari, Leo PP9 PP10
Matos, Gabriel OP40 EP96 EP94 OP95 EP102
Matthews, Allison EP97
Mattila, Mikko OP65
Matuszewski, Lukasz OP85
Mavrogenis, Andreas F OP46 OP76

Mazda, Keyvan OP57 OP67
McClung, Anna OP61
McCormack, Damian OP53
Megaloikonomos, Panayiotis D OP76 OP46
Mehlman, Charles T OP56
Mehlman, Charles T. OP122
Mehlman, Charles T EP40
Mehlman, Charles T. OP29 EP37
Mehlman, DO, MPH, Charles T EP38
Mehlman, DO, MPH, Charles T. EP44
Meier, Rahel L. OP114
Melià Grimal, María Jesús OP9
Mencio, Gregory EP29
Mendes, António Jorge EP94
Merckoll, Else PP8
Merlini, Laura OP42
Metaxiotis, Dimitrios OP80 EP92
Mezzine Harrami, Samya EP36
Micaglio, Andrea OP130
Michalski, Mark EP4
Mignemi, Megan EP25
Miklaszewski, Krzysztof OP96
Miladi, Lotfi OP70
Milbrandt, Todd OP36 OP110
Miller, Freeman OP101 OP82
Miller, Ashley OP28
Miller, Freeman EP43 OP113 OP109
Millis, Michael OP6
Mitrofanova, Ekaterina OP20
Mitrogiannis, George PP15
Miwa, Shinji OP97
Moharamzadeh, Desiree EP16 EP28 PP17 OP93
Moist, BSN, Alice OP127
Moline, Rachel EP78
Montanari, Lucrezia EP16
Moon, Sang Young PP2
Moraleda, Luis EP32 OP22 EP100
Moraleda Novo, Luis OP12
Morris, William EP7 EP65
Morris, Andrew OP47
Morris, William EP8
Morris, William Z. OP58
Morris, William OP7
Morsy, Ahmad OP97
Mostert, Adriaan OP43
Moualeu, Amanda OP132
Moukoko, Didier EP11
Mounsey, Emily OP49
Mueske, Nicole OP107 EP50
Murphy, BS, Ryan EP22
Murphy, MS, Hamadi O EP38
Muschol, Nicole OP141
Myrissa, Anastasia EP55

Nader, Gustavo A. EP53
Nakamura, Naoyuki PP5 PP3
Napora, Joshua EP19 EP65
Narayanan, Unni EP78 EP46
Nazareth, Alexander EP50 OP107
Neves, Daniella OP103 OP106 EP5
Nguyen, Joseph OP139
Nicol, Richard EP69
Nicolaou, Nicholas OP140
Nicolaou, Nick EP9
Nikakis, Christos PP15
Nordsletten, Lars OP52
Nossov, Sarah OP119
Novak, Rostislav OP30
Nuñez, Joaquin EP100
Nwachukwu, Benedict OP23

Oštádal, Martin OP19
Ocal, Mehmet Kamil OP2
Odent, Thierry OP41 OP59 OP91 OP70
Odoni, Luca OP125
Okay, Erhan EP10 OP37
Okonski, Marek OP85
Olgun, Z. Deniz OP64
Olivier, Lauthe EP82
Ömeroglu, Hakan EP24
Onay, Tolga OP35
Ondruš, Šimon EP95
Oostenbroek, Hubert EP91
Osateerakun, Phatcharapa EP13
Otsuka, Norman EP97
Ouellet, Jean EP36

Pace, James Lee OP120
Pace, James EP41
Packer, Davida OP21
Pajulo, Olli OP65
Paley, Dror OP21
Panagopoulos, Georgios N OP46 OP76
Panou, Artemisia OP111
Panski, Avi EP75
Paonessa, Matteo OP24 EP20
Pari, Carlotta PP9 PP10
Parikh, Shital N EP37
Parikh, MD, Shital N EP38
Park, Hoon OP105
Park, Moon Seok PP2
Park, Soo-Sung PP14
Pasparakis, Dimitrios OP46 OP76
Patel, Neeraj EP60
Patikas, Dimitrios OP80
Pavone, Vito EP45
Peloschek, Philipp EP77
Pennaroli, Daniela OP60
Pérez Pérez, Alba EP33
Persson-Bunke, Måns OP102
Pesenti, Sébastien OP60
Peterlein, Christian-D. OP54
Petit, Laetitia OP41
Pezaro, Sarah EP93
Pfeifhofer, Monica OP130
Pham, Anne-Dominique OP70
Philippe, Souchet OP81 EP82
Phillips, Jonathan EP88 EP87 OP59
Pierce, William OP11
Pierce, Bill OP5
Pierre, Journeau OP1
Pincher, Beth OP51
Pithioux, Martine EP11
Plánka, Ladislav EP95
Podeszwa, David OP26 OP27
Poe-Kochert, Connie OP58
Polirsztok, Eva OP14
Pollet, Virginie OP43
Pontén, Eva OP104 EP53
Porcel Vázquez, Juan Antonio OP9
Portinaro, Nicola OP111
Portman, Mark EP41
Potenza, VITO EP2
Poul, Jan EP95
Pourquier, Didier EP11

Qorraj, Hasime EP104

Rakhman, Paviel PP12
Ramalingam, Wendy EP40
Ramo, Brandon EP25
Ramseier, Leonhard E. EP67
Razi, Ozan OP132
Reading, Isabel OP47
Reddy, Manoj OP121
Rehm, Andreas OP77 EP105
Reilly, Mark OP72 PP4
Rethlefsen, Susan EP50 OP107
Rhodes, DNP, Leslie OP127
Riad, Jacques OP45
Riccio, Anthony EP21 OP92 OP31 OP11 OP26 OP27 OP5
Riccioli, Maria EP45
Richards, Steve EP25
Richards, Benjamin EP64 OP132

Robb, John OP21
Robbins, Craig OP21
Roberts, Timothy EP69
Rodby-Bousquet, Elisabet OP102
Rodda, Jill OP108
Rodgers, Jennifer OP92
Rödl, Robert OP16 EP46
Roedl, Robert OP17
Roffino, Sandrine EP11
Rogers, Kenneth OP113 EP43 OP78 OP109 OP82 EP58
Röhrl, Stephan M OP52
Rokutov, Viktor PP19 PP18
Romanenko, Konstantin PP18
Roposch, Andreas OP98
Rosenthal, Dietmar EP93
Ross, David OP5
Rotten, MD, John OP25
Rounds, Alexis OP120
Rousset, Marie OP69
Ruiz, Nicolas EP11
Rupprecht, Martin OP141
Rusch, Emmanuel OP41
Russo, Rosa EP15
Rutz, Erich OP115 OP114
Ruzzini, Laura EP15

Sá Cardoso, Pedro OP95 EP102 EP94 EP96 OP40
Sabet, Nagy EP35 OP88 EP34
Safdieh, Gabriella OP79
Safwat Hamza, Mohammed EP35 OP88 EP34
Sage, Katherine EP40
Saito, Tomoyuki PP3 PP5
Sakkers, Ralph OP43
Sala, Debra EP97 EP59
Salcedo, Maria OP22
Sales de Gauzy, Jerome OP137 OP39 OP38
Sales de Gauzy, Jérome EP83
Saliba, Elie OP129
Salom-Taverner, Marta EP66
Salvatori, Giada PP9 PP10
Samaila, Elena Manuela PP16
Samara, Eleftheria OP42
Samba, Antoine OP69
Samchukov, Mikhail OP5
Samdani, Amer EP63
Samelis, Panagiotis PP15
Sanders, Austin OP71 EP42
Sanders, James OP138
Sanghrajka, Anish P. OP98
Sanpera, Ignacio EP66
Sapogovskiy, Andrey OP123 OP131
Saran, Neil EP36
Sasala, Lee OP7
Sawyer, MD, Jeffrey EP22 EP99 EP98 OP25 OP84
Schairer, William OP23
Scher, David OP50 OP126
Schiavon, Roberto OP130
Schiedel, Frank PP13 OP17 OP16
Schiel, Maren OP54
Schmid, Mona OP141
Schmidt, Olaf OP130
Schmidt, Mona OP136
Schneider, Eleonora EP77
Schoenecker, Jonathan EP29
Schroeder, Katherine EP37
Schur, Mathew OP119 EP42 EP49 EP41
Sees, Julieanne OP101 OP82 OP113 EP43 OP109
Segev, Eitan EP46
Selber, Paulo OP108
Sessa, Giuseppe EP45
Shaarani, Shahril OP53
Shaath, M. Kareem OP72 PP4
Shabtai, Lior EP46 OP120 EP97 OP119
Sheikh Taha, Abdel Majid EP59
Shvedovchenko, Igor EP84
Silberman, Jason OP79 OP139
Silva, Selina OP119
Silva, Sara Melissa EP102
Simanovski, Naum EP75
Simantira, Ermioni OP80
Sink, Ernest OP50 OP126
Sirkin, Michael PP4 OP72
Skaggs, David EP42 EP49
Skaggs, David L. OP28
Skaggs, David OP62 OP71 OP63 OP61
Skalli, Wafa EP56 OP66 EP54
Slavin, MD, James A. EP44
Smith, John EP63
So, Conan OP23
Sofulu, Omer OP37 EP10
Solé Florensa, Pau OP87
Soler Palacín, Pere OP9
Solheim, Martine EP80
Son-Hing, Jochen EP19
Son-Hing, Jochen P. OP58
Son-Hing, Jochen EP27
Sonne-Holm, Stig EP72
Soroceanu, Alexandra EP18
Sousa, Ted OP63 EP50 OP71 OP107
Spence, MD, David EP99 EP98
Spiegelberg, Ben EP48
Spine Study Group, Children's EP63
Sponseller, Paul OP62 OP59
Spyropoulou, Vasiliki OP42
St.George, Justine OP44
Stanovici, Julien OP91
Stans, Anthony OP36 OP110
Stebbins, Julie OP4
Steen, Harald OP90
Steiger, Christina EP12 OP32
Stücker, Ralf OP141
Studer, Kathrin OP115
Study Group, Growing Spine OP62
Stutz, Christopher EP29
Sucato, Daniel OP31
Sung, Ki Hyuk PP2
Surace, Michele F EP103
Syvänen, Johanna OP65
Szponder, Tomasz OP8
Szwed, Aleksander EP61 EP57

Takeuchi, Akihiko OP97
Tamai, Junichi OP56
Tan, Sharon EP30
Taunton, Michael OP110
Tay, Guan Tzu OP73
Taylor, Daveda OP101
Terjesen, Terje EP14 PP8
Testa, Gianluca EP45
Thacker, Mihir OP78 EP58
Tharakan, Sasha EP67
Theologis, Tim OP4
Thevenin Lemoine, Camille OP137 OP38
Thevenin-Lemoine, Camille EP83
Thomas, Joanna OP47
Thomas, Wirth OP18
Thompson, George EP65
Thompson, George H. OP58
Thompson, George EP19
Tokyay, Abbas OP35 EP10
Tolaj, Arber EP104
Tolo, Vernon OP63
Topkar, Mert Osman OP35 OP37 EP10
Tran, Dong EP25
Tretow, Henning OP17
Tserovsky, Stephan PP7
Tsioulas, Paschalis EP92
Tsuchiya, Hiroyuki OP97

Tsukanaka, Masako OP52
Tuca, Maria OP139

Uglow, Michael PP6
Uiterwaal, Cuno OP43
Uppstrom, Tyler OP139
Uquillas, Carlos EP60
Urbášek, Karel EP95
Ursei, Monica OP137

Valeska, Bidault OP81
Välipakka, Jarmo OP65
van de Sande, Michiel EP91 OP43
van den Bekerom, Michel OP118
van der Lugt, Joris EP91
van Nieuwenhoven, Christianne OP133
Vaquero Picado, Alfonso EP47 OP12
Vara, Isabel EP66
Vasilescu, Dana EP81
Vavken, Patrick OP114 OP115
Vena, Giuseppe EP39
Veronese, Marcella OP130
Vial, Julie OP38
Vialle, Raphael OP134
Vidal, Christophe OP57
Villard, Li EP52
Vitale, Mochael EP63
Vittorelli, Federica OP130
Vittoria, Francesca OP125
Vlachou, Maria OP112
Vogt, Bjoern PP13 OP16 OP17
von Walden, Ferdinand EP53 OP104
Vottis, Christos OP46 OP76

Wagner, Noémie OP42
Wall, MD, Eric J EP38
Walton, Roger EP69 EP86
Wang, Ting-Ming OP124
Warner Jr., MD, William EP98
Watts, Chad OP110 OP36
Wedge, John OP49
Weerasopone, Soarawit EP13
Wehtje, Henrik OP83
Weinberg, Douglas EP8 OP7
Weinberg, Annelie EP55
Weinberg, Douglas OP6
Weir, Shannon EP78
Weiss, Jennifer EP41
Westacott, Daniel OP117 OP116
Westhoff, Bettina EP93
Whitlock, Patrick OP28
Wicart, Philippe OP134
Widmann, Roger OP50 OP126
Wientroub, Shlomo EP46
Wiig, Ola PP8
Wilczynski, Michal OP85
Wiley, Marcel OP92
Wilke, Benjamin OP36
Williams, Amy OP119
Williams MD, Nadine OP99
Williamson, Drew OP6
Wimberly, Robert OP5
Wimberly, Robert Lane EP21
Wimberly, Robert OP92 OP31 OP11 OP27 OP26
Windhager, Reinhard EP77
Wirth, Thomas EP103 OP33 OP54 EP73
Witbreuk, Melinda EP85 OP43
Wong, Christian EP72
Woo, Chin Yee OP73
Woon, Regina EP97
Wren, Tishya EP50 OP107
Wright, Patrick EP87
Wu, Kuan-Wen OP124
Wyles, Cody OP110

Yaacoub, Jean-Jacques EP56
Yahya, Ayesha OP56
Yakovleva, Veronica EP84
Yamamoto, Norio OP97
Yared, Fares OP66
Yasin, Mohamad OP61 EP18
Yaygingul, Rahime OP2
Yazici, Muharrem OP64
Yellin, Joseph OP28
Yelling, BA, Joseph OP25
Yilmaz, Serdar EP3
Yoo, Won Joon OP94
Yoon, Richard EP97

Zambakides, Christos OP46
Zambakidis, Christos PP15
Zavatsky, Amy OP4
Zelenty, William EP97
Zethraeus, Britt-Marie OP104
Zilkens, Christoph EP93

Open Access

This article is distributed under the terms of the Creative Commons Attribution 4.0 International License (http://creativecommons.org/licenses/by/4.0/), which permits unrestricted use, distribution, and reproduction in any medium, provided you give appropriate credit to the original author(s) and the source, provide a link to the Creative Commons license, and indicate if changes were made.

